# The genome sequence of the Elbow-stripe Grass-veneer, *Agriphila geniculea* (Haworth, 1811)

**DOI:** 10.12688/wellcomeopenres.18910.1

**Published:** 2023-02-17

**Authors:** Douglas Boyes, University of Oxford and Wytham Woods Genome Acquisition Lab, Darwin Tree of Life Barcoding collective, Wellcome Sanger Institute Tree of Life programme, Wellcome Sanger Institute Scientific Operations: DNA Pipelines collective, Tree of Life Core Informatics collective, James Hammond, Darwin Tree of Life Consortium

**Affiliations:** 1 UK Centre for Ecology and Hydrology, Wallingford, Oxfordshire, UK; 2 University of Oxford, Oxford, Oxfordshire, UK

**Keywords:** Agriphila geniculea, Elbow-stripe Grass-veneer, genome sequence, chromosomal, Lepidoptera

## Abstract

We present a genome assembly from an individual female *Agriphila geniculea* (the Elbow-stripe Grass-veneer; Arthropoda; Insecta; Lepidoptera; Crambidae). The genome sequence is 781.6 megabases in span. Most of the assembly is scaffolded into 30 chromosomal pseudomolecules, including the Z and W sex chromosomes. The mitochondrial genome has also been assembled and is 15.4 kilobases in length. Gene annotation of this assembly on Ensembl identified 22,132 protein coding genes.

## Species taxonomy

Eukaryota; Metazoa; Ecdysozoa; Arthropoda; Hexapoda; Insecta; Pterygota; Neoptera; Endopterygota; Lepidoptera; Glossata; Ditrysia; Pyraloidea; Crambidae; Crambinae; *Agriphila*; *Agriphila geniculea* (Haworth, 1811) (NCBI:txid1660579).

## Background

*Agriphila geniculea* (Haworth, 1811) is a moth in the family Crambidae, characterised by strong elbow-like markings across the forewing. It is found commonly across Britain and Ireland, and is particularly widespread in southern Britain, becoming more local north to the Outer Hebrides; it is primarily coastal in Ireland ( Emmet *et al.*, no date; Parsons & Davis, 2018). Globally, the moth is restricted to western Europe, extending south to the Iberian Peninsula, and east to Poland, being replaced by the closely related species *Agriphila tolli* in the Balkan Peninsula, Italy, and East Mediterranean ( Garre *et al.*, 2021; GBIF Secretariat, 2021a; GBIF Secretariat, 2021b; Lafranchis & Stefanescu, 2020; Maroń & Larysz, 2020; Plant & Jakšić, 2018).

In Britain, the moth is most commonly encountered in gardens and dry grasslands, and can be extremely abundant on coastal sand dunes. The larvae are known to feed on grasses, dwelling in a silken tube at the base of the stems ( Goater *et al.*, 1986; Parsons & Davis, 2018). Like other members of its genus, the eggs are presumed to be non-adhesive and deposited over the foodplant by the female in flight ( Léger *et al.*, 2019). This egg-laying behaviour of the females is shared by other members of the Crambinae subfamily whose larvae feed at the base of grasses, and is associated with modifications to the female genital morphology ( Léger *et al.*, 2019).

Adults of this species are on the wing from July to October and are attracted to light ( Goater *et al.*, 1986; Parsons & Davis, 2018). During the flight season adults are readily disturbed by day from grass, but Goater notes that bushes of young conifers are also a favoured daytime resting place for the adult ( Goater *et al.*, 1986).

The genome of *A. geniculea* was sequenced as part of the Darwin Tree of Life Project, a collaborative effort to sequence all named eukaryotic species in the Atlantic Archipelago of Britain and Ireland. Here we present a chromosomally complete genome sequence for *A. geniculea*, based on one female specimen from Wytham Woods, Oxfordshire, UK.

## Genome sequence report

**Figure 1.**
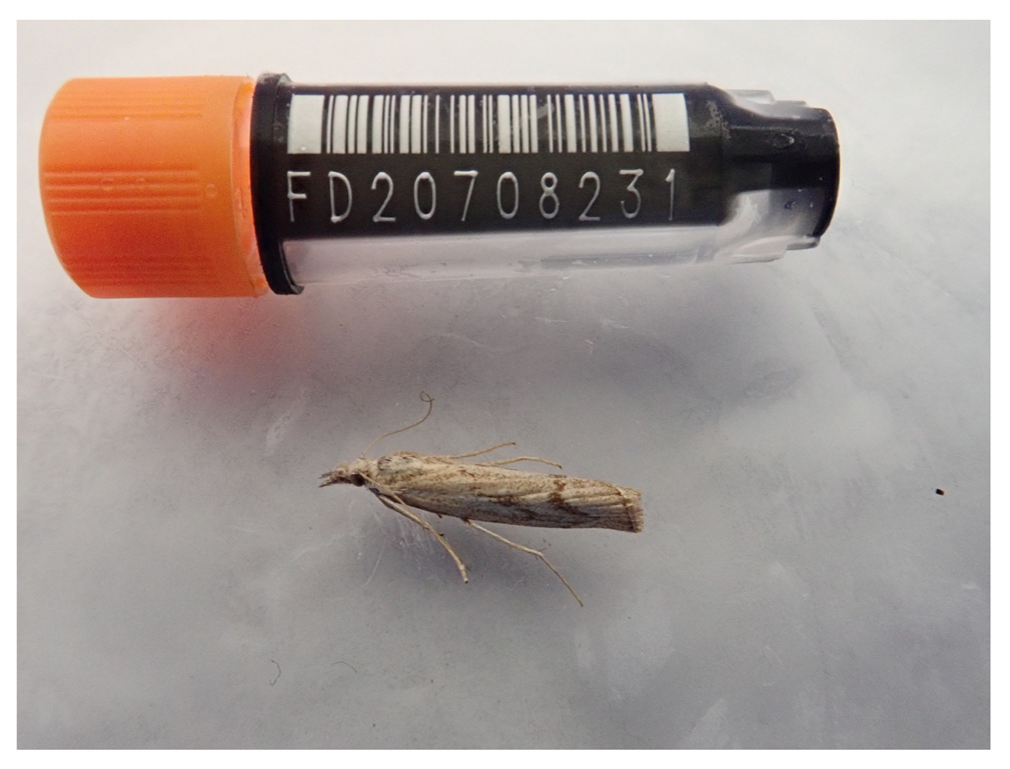
Photograph of the *Agriphila geniculea* (ilAgrGeni1) specimen used for genome sequencing.

The genome was sequenced from a single female *A. geniculea* ( Figure 1) collected from Wytham Woods (latitude 51.77, longitude –1.34). A total of 26-fold coverage in Pacific Biosciences single-molecule HiFi long reads and 59-fold coverage in 10X Genomics read clouds were generated. Primary assembly contigs were scaffolded with chromosome conformation Hi-C data. Manual assembly curation corrected 111 missing or mis-joins and removed 40 haplotypic duplications, reducing the assembly length by 1.69% and the scaffold number by 50.81%, and increasing the scaffold N50 by 1.04%.

**Figure 2.**
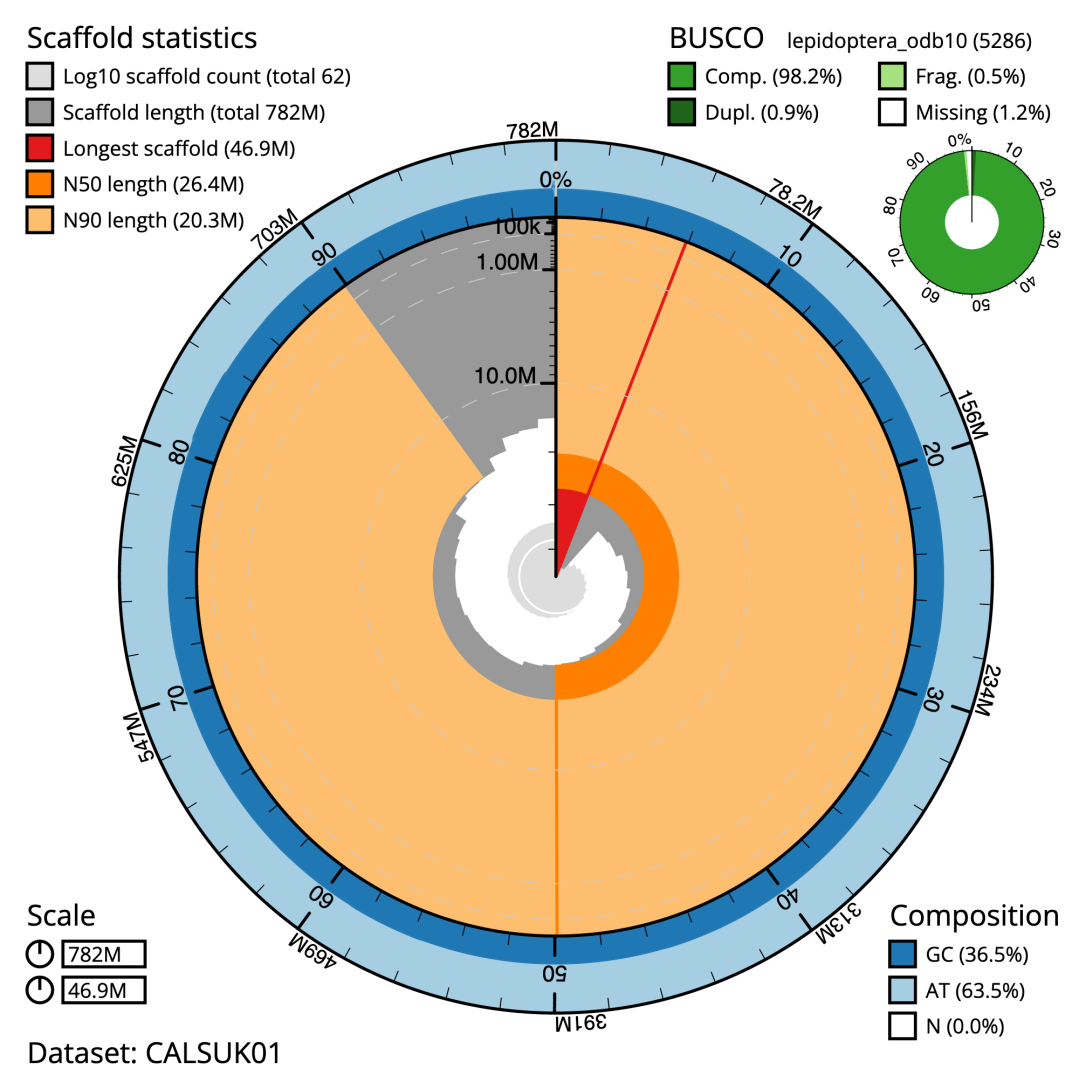
Genome assembly of *Agriphila geniculea*, ilAgrGeni1.1: metrics. The BlobToolKit Snailplot shows N50 metrics and BUSCO gene completeness. The main plot is divided into 1,000 size-ordered bins around the circumference with each bin representing 0.1% of the 781,653,547 bp assembly. The distribution of scaffold lengths is shown in dark grey with the plot radius scaled to the longest scaffold present in the assembly (46,870,843 bp, shown in red). Orange and pale-orange arcs show the N50 and N90 scaffold lengths (26,408,729 and 20,251,169 bp), respectively. The pale grey spiral shows the cumulative scaffold count on a log scale with white scale lines showing successive orders of magnitude. The blue and pale-blue area around the outside of the plot shows the distribution of GC, AT and N percentages in the same bins as the inner plot. A summary of complete, fragmented, duplicated and missing BUSCO genes in the lepidoptera_odb10 set is shown in the top right. An interactive version of this figure is available at https://blobtoolkit.genomehubs.org/view/ilAgrGeni1.1/dataset/CALSUK01/snail.

The final assembly has a total length of 781.6 Mb in 61 sequence scaffolds with a scaffold N50 of 26.4 Mb ( Table 1). Most (99.86%) of the assembly sequence was assigned to 30 chromosomal-level scaffolds, representing 28 autosomes and the Z and W sex chromosomes. Chromosome-scale scaffolds confirmed by the Hi-C data are named in order of size ( Figure 2– Figure 5; Table 2). Some collinearity between Z and W chromosomes can be observed on the Hi-C map. The assembly has a BUSCO v5.3.2 ( Manni *et al.*, 2021) completeness of 98.2% (single 97.3%, duplicated 0.9%) using the lepidoptera_odb10 reference set ( *n* = 5,286). While not fully phased, the assembly deposited is of one haplotype. Contigs corresponding to the second haplotype have also been deposited.

**Table 1.** Genome data for *Agriphila geniculea*, ilAgrGeni1.1.

Project accession data		
Assembly identifier	AgrGeni1.1	
Species	*Agriphila geniculea*	
Specimen	AgrGeni1	
NCBI taxonomy ID	1660579	
BioProject	PRJEB51038	
BioSample ID	SAMEA8603180	
Isolate information	female; whole organism	
Assembly metrics *		*Benchmark*
Consensus quality (QV)	56	*≥ 50*
*k*-mer completeness	99.99%	*≥ 95%*
BUSCO **	C:98.2%[S:97.3%,D:0.9%], F:0.5%,M:1.2%,n:5286	*C ≥ 95%*
Percentage of assembly mapped to chromosomes	99.86%	*≥ 95%*
Sex chromosomes	ZW	*localised homologous pairs*
Organelles	Mitochondrial genome assembled	*complete single alleles*
Raw data accessions		
PacificBiosciences SEQUEL II	ERR8978464	
10X Genomics Illumina	ERR8702827–ERR8702830	
Hi-C Illumina	ERR8702826	
Genome assembly		
Assembly accession	GCA_943789525.1	
*Accession of alternate haplotype*	GCA_943789515.1	
Span (Mb)	781.6	
Number of contigs	253	
Contig N50 length (Mb)	7.3	
Number of scaffolds	61	
Scaffold N50 length (Mb)	26.4	
Longest scaffold (Mb)	46.9	
**Genome annotation**		
Number of protein-coding genes	22,132	
Number of gene transcripts	22,347	

* Assembly metric benchmarks are adapted from column VGP-2020 of “Table 1: Proposed standards and metrics for defining genome assembly quality” from ( Rhie *et al.*, 2021). ** BUSCO scores based on the lepidoptera_odb10 BUSCO set using v5.3.2. C = complete [S = single copy, D = duplicated], F = fragmented, M = missing, n = number of orthologues in comparison. A full set of BUSCO scores is available at https://blobtoolkit.genomehubs.org/view/ilAgrGeni1.1/dataset/CALSUK01/busco.

**Figure 3.**
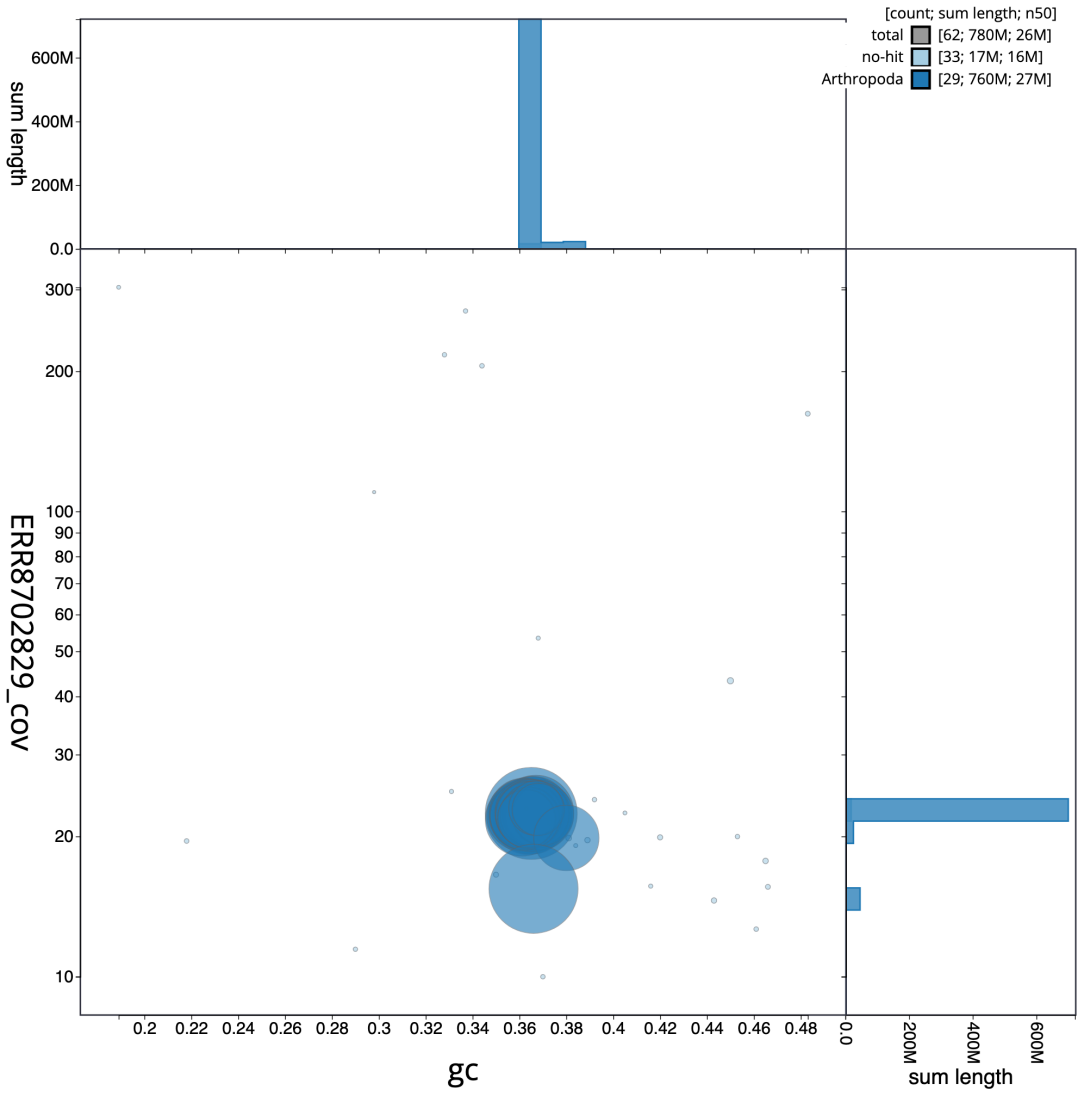
Genome assembly of *Agriphila geniculea*, ilAgrGeni1.1: GC coverage. BlobToolKit GC-coverage plot. Scaffolds are coloured by phylum. Circles are sized in proportion to scaffold length. Histograms show the distribution of scaffold length sum along each axis. An interactive version of this figure is available at https://blobtoolkit.genomehubs.org/view/ilAgrGeni1.1/dataset/CALSUK01/blob.

**Figure 4.**
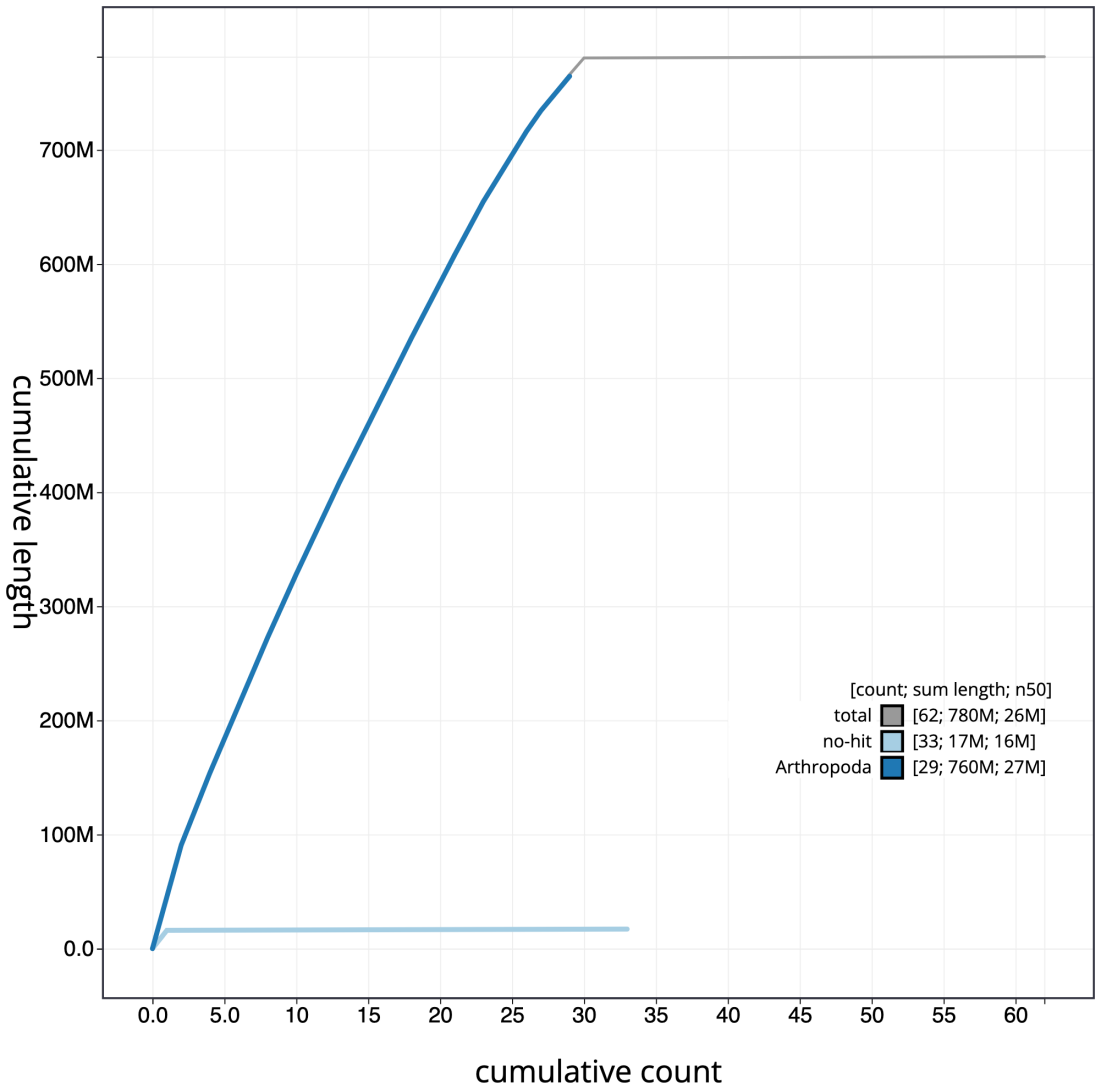
Genome assembly of *Agriphila geniculea*, ilAgrGeni1.1: cumulative sequence. BlobToolKit cumulative sequence plot. The grey line shows cumulative length for all scaffolds. Coloured lines show cumulative lengths of scaffolds assigned to each phylum using the buscogenes taxrule. An interactive version of this figure is available at https://blobtoolkit.genomehubs.org/view/ilAgrGeni1.1/dataset/CALSUK01/cumulative.

**Figure 5.**
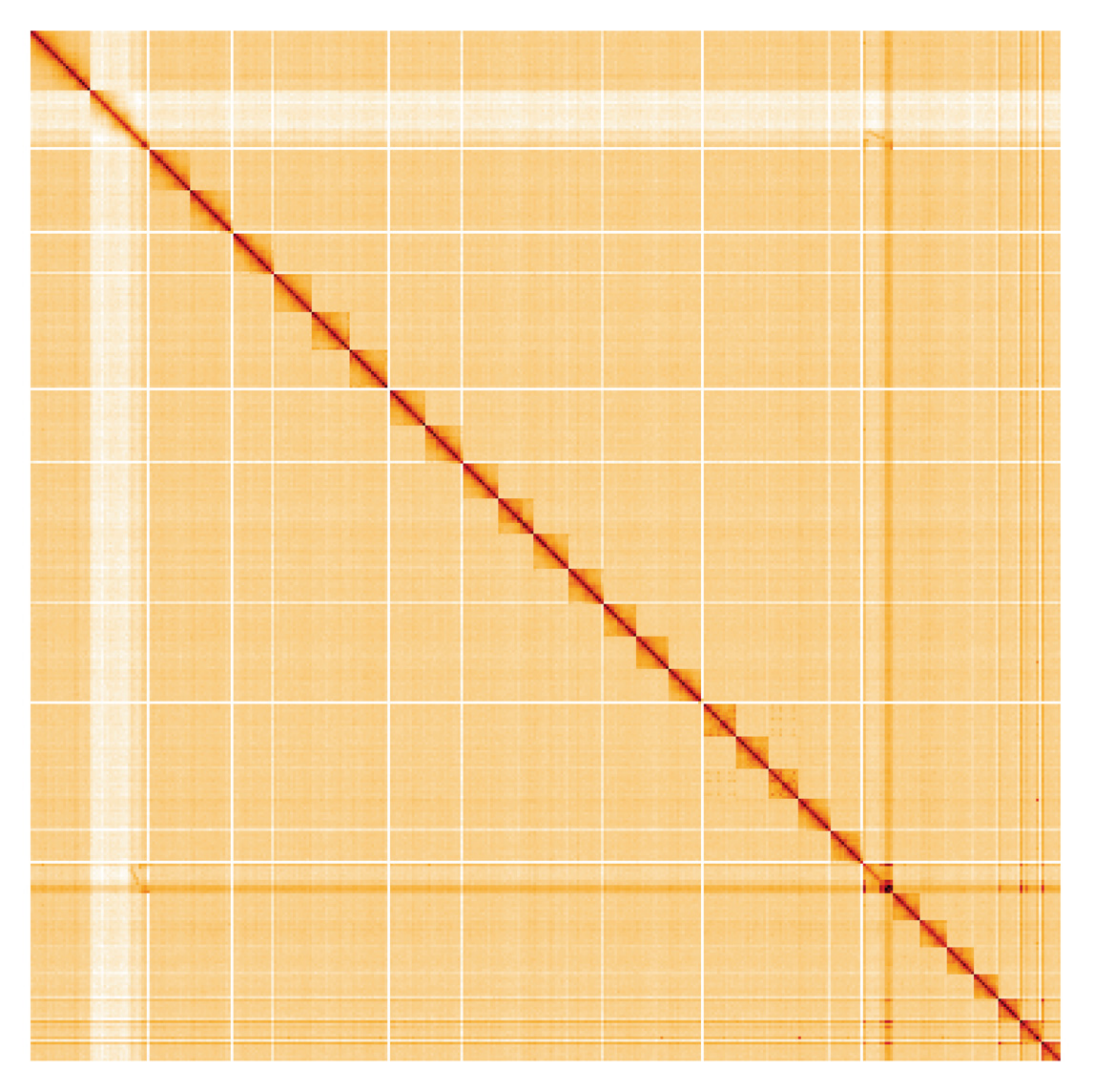
Genome assembly of *Agriphila geniculea*, ilAgrGeni1.1: Hi-C contact map. Hi-C contact map of the ilAgrGeni1.1 assembly, visualised using HiGlass. Chromosomes are shown in order of size from left to right and top to bottom. An interactive version of this figure may be viewed at https://genome-note-higlass.tol.sanger.ac.uk/l/?d=WsxgEuQCSVext3at9i4KAA.

**Table 2.** Chromosomal pseudomolecules in the genome assembly of *Agriphila geniculea*, ilAgrGeni1.

INSDC accession	Chromosome	Size (Mb)	GC%
OX038869.1	1	46.87	36.5
OX038871.1	2	31.93	36.4
OX038872.1	3	31.58	36.7
OX038873.1	4	30.25	36.5
OX038874.1	5	29.86	36.5
OX038875.1	6	29.08	36.1
OX038876.1	7	28.92	36.6
OX038877.1	8	27.96	36.2
OX038878.1	9	27.95	36.4
OX038879.1	10	27.25	36.5
OX038880.1	11	26.72	36.1
OX038881.1	12	26.41	36.2
OX038882.1	13	26.09	36.2
OX038883.1	14	25.23	36.8
OX038884.1	15	25.18	36.2
OX038885.1	16	25.03	36.4
OX038886.1	17	24.87	36.5
OX038887.1	18	24.45	36.4
OX038888.1	19	24.12	36.8
OX038889.1	20	24.1	36.7
OX038890.1	21	23.67	36.4
OX038892.1	22	20.92	36.5
OX038893.1	23	20.38	37
OX038894.1	24	20.25	36.4
OX038895.1	25	18.07	36.3
OX038896.1	26	16.04	36.4
OX038897.1	27	15.78	36.7
OX038898.1	28	14.51	36.8
OX038891.1	W	23.21	38
OX038870.1	Z	43.92	36.6
OX038899.1	MT	0.02	19.1

## Genome annotation report

The *A. geniculea* GCA_943789525.1 genome assembly was annotated using the Ensembl rapid annotation pipeline ( Table 1; Ensembl accession number GCA_943789525.1). The resulting annotation includes 22,347 transcribed mRNAs and 22,132 protein-coding genes.

## Methods

### Sample acquisition and nucleic acid extraction

A female *A. geniculea* (ilAgrGeni1) was collected in Wytham Woods, Oxfordshire (biological vice-county: Berkshire), UK (latitude 51.77, longitude –1.34) on 9 September 2020 using a light trap. Douglas Boyes (University of Oxford) collected and identified the specimen. The specimen was snap-frozen on dry ice.

DNA was extracted at the Tree of Life laboratory, Wellcome Sanger Institute. The ilAgrGeni1 sample was weighed and dissected on dry ice with tissue set aside for Hi-C sequencing. Whole organism tissue was disrupted using a Nippi Powermasher fitted with a BioMasher pestle. High molecular weight (HMW) DNA was extracted using the Qiagen MagAttract HMW DNA extraction kit. Low molecular weight DNA was removed from a 20-ng aliquot of extracted DNA using 0.8X AMpure XP purification kit prior to 10X Chromium sequencing; a minimum of 50 ng DNA was submitted for 10X sequencing. HMW DNA was sheared into an average fragment size of 12–20 kb in a Megaruptor 3 system with speed setting 30. Sheared DNA was purified by solid-phase reversible immobilisation using AMPure PB beads with a 1.8X ratio of beads to sample to remove the shorter fragments and concentrate the DNA sample. The concentration of the sheared and purified DNA was assessed using a Nanodrop spectrophotometer and Qubit Fluorometer and Qubit dsDNA High Sensitivity Assay kit. Fragment size distribution was evaluated by running the sample on the FemtoPulse system.

### Sequencing

Pacific Biosciences HiFi circular consensus and 10X Genomics read cloud DNA sequencing libraries were constructed according to the manufacturers’ instructions. DNA sequencing was performed by the Scientific Operations core at the WSI on Pacific Biosciences SEQUEL II (HiFi) and Illumina NovaSeq 6000 (10X) instruments. Hi-C data were also generated from tissue from ilAgrGeni1 using the Arima v2 kit and sequenced on the Illumina NovaSeq 6000 instrument.

### Genome assembly

Assembly was carried out with Hifiasm ( Cheng *et al.*, 2021) and haplotypic duplication was identified and removed with purge_dups ( Guan *et al.*, 2020). One round of polishing was performed by aligning 10X Genomics read data to the assembly with Long Ranger ALIGN, calling variants with freebayes ( Garrison & Marth, 2012). The assembly was then scaffolded with Hi-C data ( Rao *et al.*, 2014) using ( Zhou *et al.*, 2022)). The assembly was checked for contamination as described previously ( Howe *et al.*, 2021). Manual curation was performed using HiGlass ( Kerpedjiev *et al.*, 2018) and Pretext ( Harry, 2022). The mitochondrial genome was assembled using MitoHiFi ( Uliano-Silva *et al.*, 2022), which performed annotation using MitoFinder ( Allio *et al.*, 2020). The genome was analysed and BUSCO scores were generated within the BlobToolKit environment ( Challis *et al.*, 2020). Table 3 contains a list of all software tool versions used, where appropriate.

**Table 3.** Software tools and versions used.

Software tool	Version	Source
BlobToolKit	3.5.0	Challis *et al.*, 2020
freebayes	1.3.1-17-gaa2ace8	Garrison & Marth, 2012
Hifiasm	0.15.3	Cheng *et al.*, 2021
HiGlass	1.11.6	Kerpedjiev *et al.*, 2018
Long Ranger ALIGN	2.2.2	https://support.10xgenomics.com/genome-exome/software/pipelines/latest/advanced/other-pipelines
MitoHiFi	2	Uliano-Silva *et al.*, 2022
PretextView	0.2	Harry, 2022
purge_dups	1.2.3	Guan *et al.*, 2020
YaHS	1.0	Zhou *et al.*, 2022

### Genome annotation

The BRAKER2 pipeline ( Brůna *et al.*, 2021) was used in the default protein mode to generate annotation for the *A. geniculea* assembly (GCA_943789525.1) in Ensembl Rapid Release.

### Ethics/compliance issues

The materials that have contributed to this genome note have been supplied by a Darwin Tree of Life Partner. The submission of materials by a Darwin Tree of Life Partner is subject to the Darwin Tree of Life Project Sampling Code of Practice. By agreeing with and signing up to the Sampling Code of Practice, the Darwin Tree of Life Partner agrees they will meet the legal and ethical requirements and standards set out within this document in respect of all samples acquired for, and supplied to, the Darwin Tree of Life Project. Each transfer of samples is further undertaken according to a Research Collaboration Agreement or Material Transfer Agreement entered into by the Darwin Tree of Life Partner, Genome Research Limited (operating as the Wellcome Sanger Institute), and in some circumstances other Darwin Tree of Life collaborators.

## Data Availability

European Nucleotide Archive: Agriphila geniculea (elbow-stripe grass-veneer). Accession number PRJEB51038; https://identifiers.org/ena.embl/PRJEB51038. ( Wellcome Sanger Institute, 2022) The genome sequence is released openly for reuse. The *Agriphila geniculea* genome sequencing initiative is part of the Darwin Tree of Life (DToL) project. All raw sequence data and the assembly have been deposited in INSDC databases. Raw data and assembly accession identifiers are reported in Table 1.

## Author information

Members of the University of Oxford and Wytham Woods Genome Acquisition Lab are listed here: https://doi.org/10.5281/zenodo.4789928.

Members of the Darwin Tree of Life Barcoding collective are listed here: https://doi.org/10.5281/zenodo.4893703.

Members of the Wellcome Sanger Institute Tree of Life programme are listed here: https://doi.org/10.5281/zenodo.4783585.

Members of Wellcome Sanger Institute Scientific Operations: DNA Pipelines collective are listed here: https://doi.org/10.5281/zenodo.4790455.

Members of the Tree of Life Core Informatics collective are listed here: https://doi.org/10.5281/zenodo.5013541.

Members of the Darwin Tree of Life Consortium are listed here: https://doi.org/10.5281/zenodo.4783558.
