# Supplementary material for: The genome sequence of the Elbow-stripe Grass-veneer, Agriphila geniculea (Haworth, 1811)
Source: Wellcome Open Res. 2023 Feb 17;8:86. [Version 1] doi: 10.12688/wellcomeopenres.18910.1 (PMC10230176; doi:10.12688/wellcomeopenres.18910.1)
Supplement: Peer review history

# Peer review: Reviewer response for version 1

**DOI:** 10.21956/wellcomeopenres.20967.r58693

**Published:** 2023-05-30

**Authors:** James Mallet

**Affiliations:** 1 Harvard University, Cambridge,, United States, USA

I have no particular comments on this, and the sequenced genome seems useful as far as I can tell. 

 I don't think it's part of the needs for articles like these, but I would have thought that briefly setting the new genome in phylogenetic context would be of interest. For instance, are there any other whole genome sequences of Crambidae, or of Pyralidae / Pyraloidea that are supposedly related?

Are sufficient details of methods and materials provided to allow replication by others?

Yes

Is the rationale for creating the dataset(s) clearly described?

Yes

Are the datasets clearly presented in a useable and accessible format?

Yes

Are the protocols appropriate and is the work technically sound?

Yes

Reviewer Expertise:

Genomics, but more of a managerial role, and because of an interest in evolutionary biology

I confirm that I have read this submission and believe that I have an appropriate level of expertise to confirm that it is of an acceptable scientific standard.

# Peer review: Reviewer response for version 1

**DOI:** 10.21956/wellcomeopenres.20967.r55086

**Published:** 2023-03-13

**Authors:** Sean T. S. Law

**Affiliations:** 1 School of Life Sciences, Simon F.S. Li Marine Science Laboratory, State Key Laboratory of Agrobiotechnology, The Chinese University of Hong Kong, Hong Kong, China

This article presents the nuclear and mitochondrial genomes of the Elbow-stripe Grass-veneer, *Agriphila geniculea*. Sequenced from a female individual, the PacBio HiFi reads, 10X Genomics linked-reads and Hi-C data facilitated a final assembly of 781.6 Mb in size, most of which were scaffolded into 30 chromosomal pseudomolecules, including the Z and W sex chromosomes. As a genome note, this article described high standard tools in genome assembly and assessment. Of note, the number of autosomes assembled from *A*. *geniculea* is 28, which is different to other crambids (Chen et al., 2022; Law et al., 2022; Xu et al., 2022) ^1^^,^^2^^,^^3^. A validation step with macrosynteny against other crambids is recommended to make further proof for the completeness of the assembly, although only 0.14% of the final assembly were not anchored to any chromosome. Therefore, this assembly provides useful genomic resources for further investigations in the evolution of lepidopterans - potentially about the chromosome evolution in the species-rich family Crambidae.

Are sufficient details of methods and materials provided to allow replication by others?

Yes

Is the rationale for creating the dataset(s) clearly described?

Yes

Are the datasets clearly presented in a useable and accessible format?

Yes

Are the protocols appropriate and is the work technically sound?

Yes

Reviewer Expertise:

Evolutionary biology, population genomics

I confirm that I have read this submission and believe that I have an appropriate level of expertise to confirm that it is of an acceptable scientific standard.

## References

[1] : A chromosome-level genome assembly of Paracymoriza distinctalis (Lepidoptera: Crambidae: Acentropinae). *Arch Insect Biochem Physiol* .2022;110(2) : 10.1002/arch.21883 e21883 10.1002/arch.21883 35294789

[2] : Chromosomal-level reference genome of the moth Heortia vitessoides (Lepidoptera: Crambidae), a major pest of agarwood-producing trees. *Genomics* .2022;114(4) : 10.1016/j.ygeno.2022.110440 110440 10.1016/j.ygeno.2022.110440 35905835

[3] : Chromosome-level genome assembly of an agricultural pest, the rice leaffolder Cnaphalocrocis exigua (Crambidae, Lepidoptera). *Mol Ecol Resour* .2022;22(1) : 10.1111/1755-0998.13461 307-318 10.1111/1755-0998.13461 34228883
